# Supplementary figures and images for: Complementary mechanisms create direction selectivity in the fly
Source: eLife. 2016 Aug 9;5:e17421. doi: 10.7554/eLife.17421 (PMC4978522; doi:10.7554/eLife.17421)

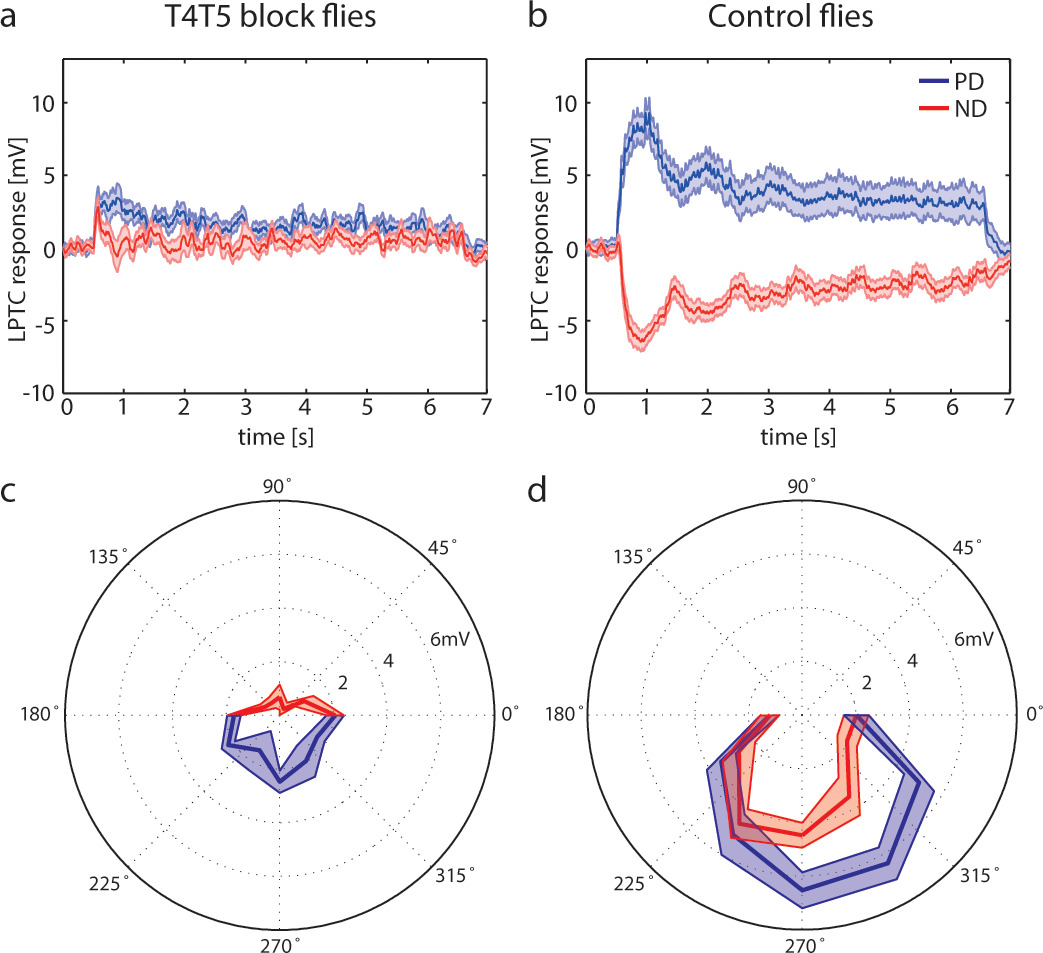

Supplement: Supplementary file 1. — (a,b) Responses of tangential cells of the Vertical System (VS) to square-wave gratings moving in the preferred (blue) and null (red) direction of T4T5 block (A, w-; R59E08-AD / UAS-TNT-E; R42F06-DBD / VT50384-lexA, lexAop-GCaMP6m; n = 5 cells in 3 flies) and control flies (B, w-; R59E08-AD / cyo; R42F06-DBD / VT50384-lexA, lexAop-GCaMP6m; n = 9 cells in 5 flies). (c,d) Directional tuning of the same cells to square-wave gratings moving in 12 different directions. Preferred direction (blue) and null direction (red) responses were averaged over the first second of stimulation. Negative null direction responses were plotted on the opposite polar coordinates. Stimuli were presented on an LED arena (see Materials and methods). Gratings had a spatial wavelength of 24°, a contrast of 1 and moved at 24°/s, i.e. at a temporal frequency of 1 Hz. In all panels, data represent the mean ± SEM. DOI: http://dx.doi.org/10.7554/eLife.17421.009 [file elife-17421-supp1.jpg]
